# Resettlement Stressors for Women of Refugee Background Resettled in Regional Australia

**DOI:** 10.3390/ijerph18083942

**Published:** 2021-04-09

**Authors:** Clare Hawkes, Kimberley Norris, Janine Joyce, Douglas Paton

**Affiliations:** 1 School of Health and Human Sciences, Casuarina Campus, Charles Darwin University, Darwin, NT 0810, Australia; Janine.Joyce@cdu.edu.au (J.J.); Douglas.Paton@cdu.edu.au (D.P.); 2 School of Psychological Sciences, Sandy Bay Campus, University of Tasmania, Hobart, TAS 7001, Australia; kimberley.norris@utas.edu.au

**Keywords:** refugee, women, regional resettlement, stressors, mental health

## Abstract

Women of Refugee Background (WoRB) have been repeatedly identified as an extremely vulnerable population. Within an Australian context, WoRB are increasingly resettled to non-metropolitan locations, otherwise known as regional locations. Despite this, to date, no research has focused on the lived experience and challenges associated with the resettlement of WoRB to regional contexts. This study aimed to address this gap in the literature by investigating the resettlement experience of WoRB resettled in Tasmania—a state in Australia classified as a rural and regional location. Qualitative interviews were conducted with a group of 21 individuals (nine WoRB and 12 service providers). Thematic analysis identified four overarching themes—Communication Barriers and Lack of Fluency in English, Challenges Accessing Everyday Basic Needs, Loss of Connection to Culture of Origin and Inability to Access Mainstream Mental Health Services for Help. Participants also highlighted a number of unique gender-related vulnerabilities experienced during resettlement, which were exacerbated in regional locations due to health services being overstretched and under-resourced. Results of the current study are discussed in regard to policy and practical implications, taking into consideration the unique vulnerabilities experienced by WoRB, which, to date, are often overlooked.

## 1. Introduction

In 2019, the United Nations estimated that 79.5 million individuals had been forcibly displaced globally, with approximately 1.4 million individuals of refugee background in urgent need of resettlement [1,2]. Resettlement is the process whereby individuals of refugee background move from the country where they have sought protection to a different country where they have been granted long-term or permanent residence status [2,3]. Although resettlement holds the potential for safety, researchers have demonstrated that the process of resettlement also comes with a multitude of challenges, as individuals of refugee background are exposed to cultural contexts and social and economic environments vastly different to their country of origin [4]. These challenges not only have the potential to increase negative health and well-being outcomes, but have also been identified as having significant long-term adverse effects on the mental health of refugee populations [5,6]. Recognition of the ever-increasing need for resettlement has resulted in a call for more research to focus on the needs and experiences of refugee populations during resettlement [5].

Australia has a long history of accepting refugee and humanitarian entrants, being formally involved in the United Nations High Commissioner for Refugees (UNHCR) resettlement program since 1977 [7]. In the 2018–2019 program, 18,750 individuals of refugee background were granted resettlement in Australia [8]. However, despite this long history, the development of programs and processes required to facilitate successful resettlement is limited [9]. This has resulted in significant gaps in the availability and resourcing of appropriate support services (for example, specialist mental health services), which impacts negatively on the adaptation and integration process across metropolitan and regional locations supporting newly resettled individuals of refugee background [9].

### 1.1. Regional Resettlement in Australia

Traditionally, the vast majority of individuals of refugee background resettled in Australia are placed in major metropolitan cities, where services are more readily available and accessible [10,11]. However, over the past decade, the Australian government has facilitated and prioritised the resettlement of refugee populations to rural and regional locations [10], with the government position in 2021 aiming to have 50% of new humanitarian resettlements allocated to a regional location by 2022 [12]. In its broadest sense, ‘a regional location’ can be identified as a ‘non-metropolitan area’, or parts of Australia with a population greater than 100,000 people outside of major cities [13]. The regional resettlement of refugee populations has been argued by the government as being an opportunity to address employment shortages, create economic opportunities and promote population growth, therefore ensuring the sustainability of rural and regional towns [12]. However, there is a growing concern surrounding the lack of current knowledge regarding the resettlement experiences of individuals of refugee background in rural and regional locations of Australia [3,11,14,15], bringing into question whether rural and regional locations can provide adequate resettlement support. Urgency in the need to understand the nature/significance of this issue is magnified by government pressure to achieve its regional resettlement goal of 50% of new humanitarian resettlements allocated to a regional location within a 12–18 month period. The current research addresses this urgency by exploring the lived experience of individuals of refugee background, and how they can be managed.

### 1.2. Identified Challenges Associated with Regional Resettlement

The emerging body of research focusing on rural and regional resettlement in Australia indicates that individuals of refugee background resettled in regional locations are not only at greater risk of experiencing mental health symptomology than the general population [14,15], but experience compounded resettlement stressors including communication and language barriers [10,16], disconnection from family [16,17,18], and loneliness [14,15,16]. Loneliness and disconnection from family is further compounded by individuals without a social connection or existing family support (identified within policy as ‘unlinked’ individuals of refugee background) being directed for regional resettlement in Australia [19,20,21]. In addition, it has been identified that individuals of refugee background resettled in rural and regional Australia have significant difficulty accessing specialist support services, such as mental health services for victims of torture and trauma [17], which in turn places increased stress on ‘mainstream’ services which are already under-resourced and overstretched. These ‘mainstream’ services are also likely to lack the expertise and resources specifically required to facilitate the integration of refugee populations into the community.

Mainstream services in rural and regional Australia are experiencing a critical shortage of skilled health professionals [18], with health professionals who work in regional and rural locations reporting longer overall work hours and more clinical contact with patients per week compared with their metropolitan counterparts [19]. This results in all individuals living in regional and rural Australia having poorer access to health services [19] and increased burnout rates and high turnover of staffing [20]. These challenges in regional and rural health services, along with the lack of specialist services to support refugee population, have been identified as having potentially adverse implications in the successful integration of refugee populations [21]. Overall, this suggests that there is a significant gap between the Australian government’s plan to increase regional resettlement and the capacity for rural and regional locations to adequately support the needs of refugee populations, placing a very vulnerable population at greater risk of experiencing ongoing difficulties.

### 1.3. Women of Refugee Background Resettled in Regional Australia

One particularly vulnerable group that is increasingly represented in regional resettlement in Australia is Women of Refugee Background (WoRB) [22]. WoRB face significant risks from experiencing ongoing stressors during resettlement, including remaining vulnerable to violence and exploitation after resettlement [23], and having less access to education, resources and paid employment than their male counterparts [24]. Despite evidence for the existence of these gender-specific vulnerabilities, the differing needs of WoRB are rarely recognised within policy and practice in resettlement countries, including Australia [24]. This significant oversight identifies an urgent need for research to identify the realities of WoRB during resettlement [25,26] and how these can be proactively managed. Not doing so will result in a failure to develop effective resettlement responses and practices [14,24], increasing the risk of these gendered vulnerabilities, which are often entrenched in the patriarchal social structure of the WoRB country of origin, being perpetuated in their country of resettlement [24]. In addition to compounding the physical, adjustment and mental health issues present in an already vulnerable population, this failure will exacerbate the demands on already stretched health and support services by presenting challenges that they are currently less well-equipped to manage. To date, research investigating the gender vulnerabilities and stressors associated with the resettlement of WoRB to rural and regional locations of Australia does not exist [27]. Therefore, the current study aimed to address this notable and urgent gap within research, by exploring resettlement stressors in WoRB resettled in a regional location of Australia. This study holds the potential to inform future policy and practices to increase the likelihood of positive resettlement outcome and enhancing the well-being of WoRB during resettlement.

## 2. Materials and Methods

### 2.1. Setting

The current study was conducted in Tasmania, an island south of mainland Australia, which has a population of 509,965 [28], with rurality classifications ranging between regional and very remote. Tasmania is also the least multicultural population in Australia (80.7% of its residents being born in Australia, from European ancestry) and takes a higher proportion of humanitarian entrants relative to its overall migrant intake (25–32% of migrants arriving in Tasmania entering on humanitarian visas) [29]. Tasmania also has a large number of WoRB, with over one-quarter of the humanitarian entrants being on the ‘Women at Risk’ 204 visas, and is currently the resettlement location of over 10% of Australia’s intake of Women at Risk [22]. As such, Tasmania provides a useful context in which to examine the impacts of resettlement in rural and regional locations on WoRB.

### 2.2. Ethical Consideration

Research involving individuals of refugee backgrounds poses a number of ethical challenges [30]. In particular, informed consent is impacted by limited English fluency and comprehension and varying levels of literacy in participants’ own language [30,31]. These ethical challenges were addressed by the selection criterion (being able to speak English at a level where the interview could be conducted in English) and providing the participants with the opportunity to give either written consent, oral consent, or both (with 7 out of the 9 WoRB opting to provide oral consent to participate in this study). These considerations were ultimately implemented to increase the likelihood of the WoRB understanding the purposes of this study, the associated risks and benefits, and thus allowing them to provide informed consent. Ethics approval for the current study was obtained through the Tasmanian Social Sciences Human Research Ethics Network (H0017941 and H0020021) and Human Research Ethics Committee at Charles Darwin University (H19003; H19087).

### 2.3. Design

A qualitative methodological framework was utilised, consisting of individual semi-structured interviews guided by a set of open-ended questions (Table 1).

### 2.4. Sampling, Recruitment and Participants

Purposive sampling techniques were employed in the initial stages, with participants being consciously selected on the basis of their capacity to contribute to the research and comprised of both individuals who identified as WoRB, as well as volunteers and service providers who support WoRB. Service providers and volunteers were invited to participate in the current study as they play a key role in supporting WoRB during the early stages of resettlement, and influence factors associated psychological well-being, including meeting daily living needs and gaining awareness of, and access to, support services [32], thus they are able to provide vital insight into stressors associated with resettlement in rural and regional locations. Service providers and volunteers participating in this study could identify as male or female to participate.

Subsequent participants were identified via snowball sampling techniques. All participants needed to be over the age of 18 and speak a level of conversational English which allowed the interview to be completed in English. The location and time of the interview were determined by a conversation occurring via telephone. Interviews were conducted in sites of service delivery, participant homes and private study rooms at public libraries, with only the participant and researcher present. WoRB who agreed to participate in this study were offered a $20 gift voucher as compensation for their time. All interviews were conducted between May 2019 and August 2020. Data collection was paused between February 2020 and June 2020 due to the COVID-19 pandemic. Data collection was re-commenced in July 2020 following ethics approval. At the time of recommencement, Tasmania had not had a community acquired case of COVID-19 for 40 days, and borders were closed to other states and territories in Australia as well as international arrivals, with hotel quarantine being mandatory for all people entering the state.

A total of 21 individuals participated in the interviews—nine WoRB and 12 service providers (four individuals in volunteer-based roles and eight in paid roles who support WoRB during resettlement). Further participant demographic information was not collected, and hence is not reported in the current study. This is to ensure confidentiality and anonymity of participants and an ethical requirement, due to the regional location, and the participants having unique characteristics (i.e., being a WoRB or working/volunteering for one of the limited refugee support services), which increases the likelihood that they would be more identifiable to local stakeholders than members of the general population.

All interviews were conducted by the first author, a female clinical psychologist who had completed a two-year, Masters’ level degree in clinical psychology and two additional years of supervised practice by a Psychology Board of Australia-approved supervisor. In addition to this, the first author has over 10 years of experience working with vulnerable and culturally diverse populations locally and internationally.

Interviews ranged from 45 to 75 min in length, and were audio recorded. Each participant was interviewed once, and individually, resulting in a total of 21 interviews being conducted. No participants were known to the researchers prior to meeting, and time was spent developing rapport prior to commencing the research interview. The aims and goals of this study were explained to all participants, who were also provided with a plain-language information sheet prior to giving informed consent.

Audio-recordings from interviews were transcribed verbatim and interviewees were provided the opportunity to review the transcript for comment and/or correction.

### 2.5. Data Analysis

Data saturation, the point where no new interview themes emerged, determined the final number of interviews required for this study. To identify when saturation occurred, data were continuously analysed throughout the data collection period using Nivo 12 software. An audit trail was kept throughout the research process to aid the researcher in identifying when data saturation was reached. Within the current study, by interview 17, the audit trail entries illustrated that the list of new themes began to decline, until there were no new themes identified from the 21st interview, hence this was deemed the last interview.

As the current research focused on reporting the experiences and reality of participants, transcripts were analysed utilising the Braun and Clarke [33] six-step framework for conducting thematic analysis which employs inductive thematic description at the semantic level, underpinned by an essential/realist approach. Step one involved familiarisation with the research data. Within the current research, this involved reading and re-reading the data whilst taking notes about possible themes and codes within transcripts. Step two involved generating initial codes, in which a list of ideas and interesting themes were generated from the data. The current study employed complete coding, in which ‘anything and everything’ of interest or relevance within the dataset was coded. Step three of the analysis involved searching for themes, in which different codes were sorted into potential themes, and all data extracts associated with identified themes were collated. This was achieved by identifying potential patterns across the coded data. Step four involved reviewing and refining the themes. This involved the researchers checking the themes against the dataset to ensure they represented the dataset. Step five involved defining and naming themes and developing a detailed analysis of each theme. Step six involved the write up phase and weaving together the analytic narrative and contextualising the analysis in relation to existing literature. NVivo qualitative data analysis software (version 12) was utilised for data management. Reporting was guided by the Consolidated Criteria for Reporting Qualitative Research (COREQ) [34].

## 3. Results

Four overarching themes were identified within the data—Communication Barriers and Lack of Fluency in English, Challenges Accessing Everyday Basic Needs, Loss of Connection to Culture of Origin and Inability to Access Mainstream Mental Health Services for Help.

### 3.1. Communication Barriers and Lack of Fluency in English

Overall, communication barriers and lack of fluency in English were identified as a significant acute and chronic stressor during resettlement for WoRB which impacted their ability to express their needs and form relationships in their new communities. WoRB also face additional barriers when in terms of attending language classes, including taking on the primary caregiving role for their children, and never participating in an educational setting before, making the experience of learning a new language in a novel environment incredibly stressful. In addition to this, WoRB often arrived in the regional locations with such a low level of English that the allocated government-funded English classes were insufficient. Language and communication derived from the socio-cultural challenges of resettlement create additional challenges for meeting everyday life and livelihood needs.

The WoRB respondents emphasised the challenges and stressors associated with language, and in particular the capacity to communicate their needs in the English language, which was highlighted as a vital factor in helping them settle.

‘*I think your ability to go out and learn English is a huge factor that helps people settle in because then you’re able to communicate with people it’s a lot easier, and you’re able to fit in a lot easier*’

—WoRB 1

WoRB identified difficulty in communicating not only practical needs but also affective experiences and differing social and cultural conventions as a significant stressor, which had ongoing implications in regard to their mental health during resettlement, and limited their capacity to express their needs.

‘*It was hard, and maybe it was because of the language, and the language differences, we were not able to express how we felt, and we couldn’t tell people what we wanted*’

—WoRB 6

‘*It is very difficult, even though we can feel inside in our mind and heart, if we don’t have the language, if we don’t know what to say and what to do, it’s very very hard… and it’s kinda a mental pressure that you don’t get to express what you wanted to say to people…*’

—WoRB 7

‘*Because most of our people are, how do you say, not illiterate, but they don’t know how to speak or read English, so they find it hard … and that can also make it very stressful and they don’t know how to speak and how to talk to the teachers about their children’s progress that is happening in the school. So that can also make them feel more stressed*’

—WoRB 9

Having limited English proficiency was also expressed as having ongoing impacts in other areas of WoRB lives, including the ability to gain employment.

‘*I think the language is the most common issue that most of our people have experienced and also after going to school and getting some language skills, I think the job is also one of the biggest issues from within our community. Because even though they can actually perform the task, they can’t express that. So it all comes down to language. So even though they can perform the skill, and do all the tasks like other people do, the communication is making them behind and prevents them from getting a job*’

—WoRB 6

‘*I know a lot of the women probably don’t feel capable of being able to find work due their language barriers*’

—Service Provider 2

In regard to learning English during resettlement, WoRB expressed having additional barriers to overcome, due to childcare obligations, and limited exposure to being in an educational context prior to resettlement.

‘*They need help, but especially the women who have many kids at home… the little little kids … and they really need to have help with their English*’

—WoRB 5

‘*For me it was when I delivered my baby, for me it was hard because that kept me busy at home, yes, but for another family, they don’t have enough education, and they don’t know things like the alphabet and do not have even very simple English, so for them it is very hard*’

—WoRB 6

‘*I think learning English is one of the big ones for a lot of the women I work with; especially if they aren’t used to putting their kids in childcare. Also, I think, for some of the women I know, they’re not used to, sort of, speaking up and being assertive, so the whole classroom experience is quite traumatising for them. Like, they feel that people might be judging them, and so, I’ve worked with women who just refuse to go to English classes, because they, they just don’t feel safe in that classroom, having to speak up in front of others; and also [they] struggle, you know, to leave their children in childcare with a stranger while they go to English classes*’

—Service Provider 5

‘*There are often cultural barriers in place so that refugee women aren’t encouraged to attend English conversation classes to improve their English. Low English proficiency comes along with isolation as it can be difficult therefore to communicate with services such as Centrelink, Medicare, the bank, and navigate the health system, public transport, school enrolments, and housing*’

—Service Provider 3

Despite language being identified as one of the biggest challenges during resettlement in regional areas of Australia, particularly for WoRB due to additional barriers, service providers expressed that the current services available are not enough to address the need.

‘*We’ve got a lot of really low-level English women that have exhausted all their formal English hours, and they’re still really low-level of English… but they’re not even eligible for our programs anymore*’

—Service Provider 12

### 3.2. Challenges Accessing Everyday Basic Needs

Overall, accessing basic needs, in particular accommodation, was identified as a stressor during resettlement for WoRB. WoRB also expressed that they experienced significant financial stress due to dependence on government support and difficulty finding additional income through paid employment. This in turn caused significant distress and ongoing instability in WoRB daily lives. Due to this difficulty in accessing housing and financial security, many WoRB identified that members of their own community of origin were leaving regional areas, in hope of greater stability and support.

The WoRB interviewees emphasised that a key stressor in resettlement was performing basic everyday tasks, and having access to essential everyday needs and social connection.

‘*I needed help just getting used to doing basic things, because I would feel anxious because it’s a new place, and so you know, if someone had been there to, like, take me around, show me shops, make me feel a little more comfortable, that would have been great*’

—WoRB 1

In particular, one of the core stressors identified by WoRB was accessing stable accommodation, with many of them waiting for an allocation to an appropriate house and spending prolonged periods in crisis and temporary accommodation.

‘*Living in temporary accommodations for 12 months at the beginning was difficult. The uncertainty was very difficult*’

—WoRB 4

The lack of stability was also emphasised as a significant stressor by service providers supporting WoRB during resettlement.

‘*Our biggest challenge from a counselling perspective is the myriad of needs that come into the counselling space and they are, dare I say, in Maslow’s Hierarchy of Needs—it’s housing, it’s shelter, it’s food*’

—Service Provider 1

‘*They can’t even stabilise—stability being a big one and whether that’s accommodation, financial*’

—Service Provider 3

‘*If mums you know, a single mum, she’s overwhelmed, -the whole family is basically on a crash course towards being homeless because of the stress of housing*’

—Service Provider 12

WoRB faced particular difficulties accessing basic everyday needs due to financial stress and ongoing dependence on government support due to limited job opportunities.

‘*In Tasmania, there are not many jobs and you have to pay a lot of money*’

—WoRB 3

‘*You know a lot of the women probably don’t feel capable of being able to find work due their language barriers or confidence or just being mums and having kids to look after. Ah not having a driving licence all of those things. So there’s this I guess, worry, constant worry that they’re having to live off such a low income and really dependant on their Centrelink, and that financial stress which is just sitting in the back of their heads all the time knowing that there’s so much to do with that little amount of money*’

—Service Provider 2

‘*A lot of the women who came here on the Women At Risk visa, single mums with five or six children, I mean, trying to get a rental here, is nearly impossible. So, the stress of that … I mean, I think every day, I must be talking to someone distressed; housing is probably one of the biggest stresses for a lot of people at the moment*’

—Service Provider 5

This inability to access basic needs, such as housing and employment, resulted in WoRB relocating from regional areas to metropolitan locations.

‘*Many of them are moving because they cannot find a job*’

—WoRB 5

‘*Many of my friends, they say, I’m not supported. I’m not feel—Like this isn’t the right environment for me’ and then they move to somewhere else*’

—WoRB 8

‘*A lot of people are moving interstate because there’s a lot more flattering options for employment and housing*’

—Service Provider 4

### 3.3. Loss of Connection to Culture of Origin

Overall, WoRB identified that they experienced stressors during resettlement associated with a loss of connection to their culture of origin. WoRB expressed particular stress and concerns associated with their children losing a connection with their culture of origin, as their children developed an increase in connection to the Australian culture. Particular gender-related stressors were also identified for WoRB, due to the resettlement culture providing them with alternative options surrounding social, employment and family-based roles, and relationship options, which were traditionally dictated by their gender within their country of origin and deviated from their traditional cultural norms.

WoRB expressed the impact that a loss of connection to their culture of origin can have during resettlement, and in particular the stress associated with their children losing a connection with their culture of origin.

‘*For single women who are raising their kids on their own, who are on a Women at Risk visa, there is always a lot of stress and anxiety about them not wanting their kids to lose connection with who they are or where they’ve come from. And yeah it is hard for them to understand that their kids are growing up in a Western sort of culture now*’

—WoRB 2

‘*When the kids grow up and become teenager, they don’t like to come back to their religion and culture, and they thinking, this is for past memory and they are like ‘mum, mum, don’t think about past memory’, and I tell them, no, you must listen*’

—WoRB 5

‘*Kids forget the home language… for example, my daughter ask me what these things in [language removed], and I was like ‘My god, you forget everything*’

—WoRB 9

The concern around children losing a connection with their culture of origin was highlighted as creating intergenerational tension, and significant stressors within the family unit.

‘*It has big impacts on family dynamics, particularly for a lot of the single mums. Women [on] at risk visas have been in particularly tough situations, often they have, like quite a few children, some of them might be older boys, like older sons and then they’re struggling as well with how to settle in a new country, how to support their family, the traditional roles, maybe having to be the man of the house and maybe that is not quite working in a new country where there might be different values*’

—Service Provider 4

‘*I think there’s a lot of family conflict, I think creates a lot of mental distress for many of the women I work with, especially if they’re single parents. So it’s very challenging, the roles have changed, and if their adolescent kids, become more, you know, involved in Australian culture; so I’ve seen a lot of distressed and upset women because it’s just so challenging for them to manage that role, and in a new country*’

—Service Provider 5

These intergenerational tensions within the family unit were also emphasised as being particularly pertinent for younger WoRB, as the resettlement culture often provided them greater opportunity for development and growth than their culture of origin, which deviated from traditional expectation surrounding gender-based roles and marriage.

‘*The young people want to start to make their own choices, and have a say in who they get involved with and get married, but parents still align with those older traditions, and in accordance of keeping blood, you know, within the family, and so yeah, so I feel as though, that … what do we call it? Cultural adaptation, you know, when learning … coming into a new culture, so I think that that’s always that stress*’

—Service Provider 8

These opportunities which are afforded to younger WoRB can also cause a deeper level of conflict in older WoRB as they feel torn between their daughters following in their footsteps, and aligning with traditions in their culture of origin, and having access to more opportunities in the host country.

‘*It is interesting, seeing the mothers, you know, coming to terms with that stuff, too, because the only options for them, and what was always expected of them is, no, you marry whoever you’ve been chosen to marry; that’s your responsibility, that’s just what you need to do, to honour your family, and for them, it was like, well, there wasn’t opportunities for me to go and get a career, or study, and for them to now come to terms with that, whilst them being really keen for their kids to get [an] education. It’s a tricky, tricky space*’

—Service Provider 12

### 3.4. Inability to Access Mainstream Mental Health Services

Overall, accessing services for mental health support during resettlement was highlighted as a stressor for WoRB. It was identified that support offered via mainstream services was insufficient for WoRB due to their level of need. Mainstream services were also identified as being difficult to access due to services having limited training in working in a culturally sensitive framework. This resulted in services rejecting a referral on the basis of the women being identified as being from a refugee background, and not using interpreters, resulting in children acting as interpreters in situations which can result in vicarious trauma for the child (such as a crisis mental health presentation), or self-censorship by the parent. The ability for mainstream services to provide appropriate services to WoRB resettled in the regional location was further compounded by systematic issues, including the mental health service in general being pushed to the extreme in the regional location, due to being general under-resourced and under-funded. This in turn resulted in bi-cultural peers workers supporting WoRB with mental distress, which they felt underprepared for.

WoRB and service providers emphasised how stress in resettlement was exacerbated due to the difficulty associated with accessing mainstream services in Tasmania for support.

‘*Mainstream services here try very hard, and that’s something to be appreciated, but I feel like the bigger problems that services face here are structural, so limitations in funding. So, like, for me, when I try to access a mental health service, I only have six sessions with a service… like, with a counsellor, and once those six sessions are over, then I’m expected to [pay] out of pocket. And, six sessions is not enough for someone to address, like, all the trauma that they have experienced up to that point, if they are a refugee, or a migrant*’

—WoRB 1

‘*It’s all reactive stuff that we’re dealing with. Mainstream services really need to be skilled up. There’s been some really, really full-on situations in the last couple of years, and I’ve tried to refer people on. And as soon as they hear multicultural background, or a refugee woman, or someone who’s English is not great, they very quickly hand them back to me*’

—Service Provider 8

‘*I had to help a young person go into sheltered accommodation, and I was dealing with the services here and, trying to refer her on, it kept getting sent back to me. So, you know that was a real learning experience*’

—Service Provider 10

The difficulty in accessing mainstream services was highlighted in the interviews as stemming from larger systemic issues, including mental health services in regional areas being generally under resourced and pushed to extremes.

‘*We’ve got a pretty broken Mental Health system anyway, so, it’s not necessarily unique to the refugee cohort, so, how do they rub up against it? like it’s a system under so much stress and turnover and whatever. I would say that is a really high-risk environment for our [refugee] cohort*’

—Service Provider 12

‘*Even though they’re funded by state and federal governments, there’s a big barrier to accessing mental health supports because a lot of services just say, well, we don’t have a budget; and I’ll say, well you should have one because you’re funded by the government, but it’s not a priority to look after culturally diverse communities*’

—Service Provider 5

‘*The community services are so stretched they don’t engage. They’ll do a certain amount of effort and then they’ll close. But there’s people with like cultural, language and kind of conceptual understanding barriers, so it’s kind of predictable what happens really–they don’t get the service*’

—Service Provider 1

Mainstream services were also identified by participants as having limited training and funding in working with refugee populations, in particular having limited understanding surrounding working within a culturally sensitive framework, including providing WoRB access to interpreters.

‘*One of the biggest complaints we get from community members is that staff either don’t use an interpreter, or insist on them using family, or they don’t know how to work properly with an interpreter*’

—Service Provider 5

‘*I just think mental health services here, generally, aren’t going so well: we’re at a bit of a crisis as well, so … and, one of the biggest things is if you have a mental health issue, and you present to [service name removed], they will force your children or family members to interpret for you. They will not get you a professional, unbiased, interpreter to interpret for you*’

—Service Provider 10

‘*we’ve had cases of a suicidal mother, and her son being forced to interpret her mental health crisis. And I just think that’s criminal. So, the damage that services are doing by not using interpreters is incredible*’

—Service Provider 12

‘*that’s a really big issue here in Tasmania because we find a lot of the non-government organisations, you know, governments are outsourcing things, um, often they don’t have a budget for interpreters*’

—Service Provider 6

The inability to access mainstream services, and the lack appropriate training for workers to work in a culturally sensitive framework to support diverse groups also had significant ongoing ramifications for WoRB mental health.

‘*As a refugee woman, myself, I guess the impact of mental health is epic. It’s huge. I guess I can mostly speak from my own experience, when I arrived, I wasn’t eligible for the settlement services… and I fell through the system*’

—WoRB 2

Not being able to access mainstream mental health services, or experiencing culturally inappropriate responses resulted WoRB turning to bi-cultural and peer workers to support them with their mental distress.

‘*A bi-cultural worker plays a huge role in making the individual feel more comfortable. I have seen people expressing their issues freely and openly to a bi-cultural worker then the other people, so I have experiencing that one aswell*’

—WoRB 8

‘*having someone from the community can speak the same language as our people can make them more comfortable in opening up about their feelings and all that*’

—WoRB 6

However, this came with additional challenges for bi-cultural peer workers, as they were often not trained mental health professionals.

‘*Basically, I have not been given the training that can help us [with mental health]. So I think gaining more training in that area will play a huge role in helping bi-cultural workers think of a way that will help people from the community. I think more training should be provided*’

—WoRB 7

## 4. Discussion

To our knowledge, this is the first study to investigate resettlement stressors in WoRB resettled in a regional location of Australia [27], which addresses the dearth of research focusing on refugee populations resettled in rural and regional locations of Australia identified in earlier work [35]. This study is also novel in addressing a broader call for research to focus on the gendered factors which shape and influence the overall well-being and mental health of WoRB during resettlement [26]. The importance of the work and its identification of policy and practice issues is further reinforced by Federal government goals of increasing WoRB resettlement in regional areas by 2022. The results highlight and emphasise the increased vulnerability of WoRB during resettlement, and the lack of preparation, in terms of service capacity and community support, for the increased resettlement of refugee populations to regional and rural areas. This in turn not only increases the degree of stress experienced to the WoRB and their families, but also places significant pressure on regional and rural mental health services, which are already severely underfunded and under-resourced [36,37].

Consistent with research conducted by Watkins, et al. [38], which identified that English language proficiency and communication barriers had the largest impacts on well-being in WoRB resettled in metropolitan areas of Australia, WoRB resettled in regional Australia also emphasised the significant impacts that English language proficiency and communication barriers had on them during resettlement. Poor language proficiency has been identified as having a significant association with poor self-reported health in migrant populations, with this association being significantly greater in women across time [39] Within the current study, English language proficiency and communication barriers had a significant follow-on effect for WoRB and impacted their ability to express their needs, make social connections and gain employment. It was also identified that WoRB have additional barriers to overcome in an attempt to develop their English proficiency skills, including being at a disadvantage within a learning context. The need for language programs for refugee populations in regional areas to be flexible has been highlighted by Smith, Hoang, Reynish, McLeod, Hannah, Auckland, Slewa-Younan and Mond [3], with the current study further emphasising that flexibility, in regard to the how the language program is delivered and the amount of hours available, is paramount when supporting WoRB in an educational context. Building on the need identified by Smith, Hoang, Reynish, McLeod, Hannah, Auckland, Slewa-Younan and Mond [3], the current study emphasised the particular vulnerability of WoRB in the educational context, and that the current English program in regional areas is insufficient. This is due to many WoRB exhausting their allocated hours before having a level of English which will allow them to function in their new community day to day. The findings of this study have implications in regard to the development of a more flexible English language program which acknowledges the additional challenges that WoRB face in the educational context, including parenting responsibilities and limited prior learning experience in a formal classroom. Taking these factors into consideration will be key for future program and policy officers, as it is evident that the current program and supports do not take into consideration the gendered challenges that WoRB face in the educational context during resettlement, leaving WoRB at a particular disadvantage.

WoRB and service providers identified how the lack of access to basic needs caused significant stress during resettlement in regional areas. In particular, WoRB experienced difficulty accessing stable accommodation, experienced financial strain, and experienced difficulty finding employment, which resulted in ongoing and chronic instability. Gaining successful and stable employment has been identified as a significant indictor of successful resettlement in refugee populations [40]. Research by Curry, Smedley and Lenette [40] highlighted the difficulty faced by refugee populations resettled in regional locations of Australia in gaining stable employment, resulting in migration to larger metropolitan locations in search for employment. The current research builds on this emerging body of literature and highlights the specific gendered challenges for WoRB finding employment, due to difficulties in English language proficiency and childcare obligations. These marked difficulties in finding employment, and the adverse follow-on effects, bring into question the rationale behind the Australian government’s push for regional resettlement—namely to fill employment opportunities and create economic opportunities [12]. These findings suggest that these government policies have been rolled out without considering the additional support and funding which would be needed in these regional and rural areas to make long-term resettlement viable.

The ongoing difficulties in accessing basic needs was also identified by service providers supporting WoRB during resettlement. This included stress related to accessing food, accommodation and employment being identified as key factors for WoRB seeking services and mental health support. These findings build on previous research which has highlighted that individuals of refugee background seek support for social and practical needs over other needs such as treatment for mental health through talk therapies [41,42,43]. This aligns with theoretical frameworks including Maslow’s Hierarchy of Needs, in which physiological needs and safety needs need to be addressed before an individual’s psychological needs can be a focus [44], and Social Determinants of Health (SDoH) frameworks, which emphasise that social, political and economic factors sustain inequalities and health outcomes [5]. Stemming from the influential research by Mikkonen and Raphael [45], which synthesised and highlighted how social inequalities, in particular low income, goes hand in hand with health inequalities, research investigating the SDoH has increased significantly over the past decade [46], with the impact of SDoH on refugee populations during resettlement starting to be highlighted [5,47]. However, research investigating SDoH in regional resettlement locations is particularly non-existent [6,48]. This research addresses this gap within literature and identifies that SDoH play a significant role in the health and well-being of WoRB resettled in regional locations. The findings of this research indicate that WoRB resettled in regional locations are experiencing significant social inequalities (i.e., low income and no access to suitable housing), which in turn are having significant ramifications on their health. Future policy and program development would benefit from consulting refugee populations that have successfully resettled in a regional location to gain insight into lived experiences and what SDoH impacted on their resettlement experience, both positively and negatively. This will aid understanding of how the Australian government and service providers can proactively support integration and well-being.

WoRB identified the impact that losing a connection with their culture of origin had during resettlement. The complex process of cultural identity during the migration process has been extensively researched, stemming from the seminal work of Berry [49]. Losing connection with their culture of origin has been identified as being a significant stressor for individuals of refugee background during resettlement, and increases the likelihood of adverse mental health outcomes [50]. Furthermore, culture itself has been identified as an influential SDoH, and being denied the opportunity to engage in cultural practices has been identified as a major public health concern, particularly for migrant women, due to the negative health ramifications it can have in the long term [51]. However, it has been identified to be particularly complex in forced migrants (including refugee populations) due to the concept of involuntary migration [52]. Developing a greater understanding of the relationship between acculturation and mental health among forced migrants can be identified as paramount due to the unprecedented number of individuals of refugee background worldwide [5]. Unfortunately, there is a lack of literature investigating the topic, as it is currently uncertain whether the large body of research investigating acculturation and mental health in immigrants and racial and ethnic monitories is comparable for forced migrants [52]. Investigating this further will allow the development of more progressive government policies which protect the cultural values, practices and beliefs of newly arrived immigrants and forced migrants.

Within the current study, the stress associated with a loss of connection with their culture of origin was derived from WoRB’s concerns surrounding their children losing a connection with their culture of origin as they acculturated to the Australian culture. Children’s agency has been identified as having significant impacts on well-being during resettlement in refugee families [53], with the parent–child relationship being interdependent and bio-directional in nature [54]. Research has identified that concerns for their children is often a key factor influencing a parents decision to leave their country of origin, thus experiencing unexpected or unwanted migration outcomes, such as conflict with their children due to new parenting norms, can result in an increase in stress and have negative consequences on their health and well-being [54]. Overall, this suggests an interdependence associated with acculturation stress during resettlement between WoRB and their children [54], and highlights that acculturation models for forced migrants may be influenced by generational differences and the age of the forced migrant (for example, if they arrive as a child, or adult).

WoRB in the current study further identified that novel opportunities, including education, employment, family and marriage options, available to their gender within the country of resettlement created stress, and often tension, within the family unit. The stressors associated with divergence from traditional role expectations for daughters and wives in regard to family and marriage roles has been identified as negatively influencing WoRB experiences of depression and poor self-esteem during resettlement [55], as well as impacting engagement with mental health support services [26]. This further highlights the vulnerability and unique gendered stressors for WoRB experiencing resettlement. Overall, this suggests that greater consideration needs to be given to interventions focusing on cross-cultural social norms surrounding parenting and marriage during the initial stage of resettlement. Implementing such programs not only proactively considers the social determinates which cause many WoRB to seek mental health support during resettlement, but proactively addresses a factor which has been identified to increase the likelihood of them experiencing stress.

Finally, consistent with research by Ziersch, Miller, Baak and Mwanri [11], the current study identified significant barriers and challenges in accessing mainstream support and mental health services in regional resettlement locations in Australia, across sector, service and individual levels. On the sector level, service providers emphasised how the current state of mental health services in general in regional locations were having a negative impact on WoRB, as many of the mental health services were ‘broken’ and pushed to the extremes, resulting in WoRB falling through the gaps. This is consistent with research arguing that the mental health workforce in regional and rural locations is under-resourced and under-funded, resulting in treatment accessibility being very difficult for the general population [56]. This suggests that greater investment needs to be made into rural and regional mental health services to ensure adequate service provisions regardless of populations, which will become even more pertinent with the government’s plan to increase the resettlement numbers of this highlighted vulnerable population over the next 2 years.

On the service level, WoRB struggled to access mainstream services for support due to mainstream services having a limited understanding of working within a culturally sensitive framework This finding is consistent with previous research that has identified that individuals of refugee background have difficulty accessing mainstream mental health services due to services having limited cultural competency and responsiveness [26,41,57], which is of particular concern in rural and regional areas due to a lack of specialised services for refugee populations [17]. Due to difficulty accessing appropriate mental health support from services, the current study identified that bi-cultural/peer workers would often be a key support for WoRB experiencing mental distress, despite having no training in the area of mental health support. Bi-cultural/peer support workers have been identified as playing an important role in assisting refugee populations in regional locations access health services, as well as acting as translators and interpreters [58]. However, to date, there is limited research investigating the role of a bi-cultural workers working in the mental health space within a refugee resettlement context. This can be identified as an important area of inquiry, as it may assist in filling the gaps stemming from under-resourced mental health services in rural and regional resettlement locations, and further assist in the development and training for bi-cultural workers of refugee background in the mental health space.

The current study also highlighted the significant ramifications that having a limited understanding of working within a culturally sensitive framework can have not only on WoRB, but also on their children. In particular, participants highlighted how often children were used as interpreters for their mothers, particularly in crisis or emergency situations. This is consistent with research by Smith, Hoang, Reynish, McLeod, Hannah, Auckland, Slewa-Younan and Mond [3], which initially identified the salient use of child interpreters in regional settings in Australia, despite policies precluding the practice. The use of child interpreters in health and mental health service settings, instead of utilising official interpreting services as per policies, in regional locations has been highlighted as a form of systemic racism, and places the children at significant risk of vicarious traumatisation [3]. This suggests that further research and policy development is needed to reduce this dangerous and culturally insensitive practice from occurring in regional health services supporting not only WoRB, but culturally diverse populations in general.

The current study has several limitations, some of which are inevitable when researching minority and diverse populations. Firstly, all WoRB who participated in the current study were required to have an adequate level of conversational English, as all interviews were conducted in English. This limited the opportunity for recently resettled WoRB to participate in the interviews, as they are less likely to have developed strong enough English language skills to participate in the interview. This reduces the likelihood that the current study captured information representative of the entire resettlement process. Future research should aim to address this through the use of longitudinal designs, which would assist in determining whether stressors occurring during the resettlement process change as a function of time and phase of adaptation. The current study also used convenience sampling, which may detract from the representativeness of the sample, and therefore the overall generalisability of the findings.

## 5. Conclusions

The current study is the first to investigate resettlement stressors in WoRB resettled in a regional location of Australia and addresses a significant gap in literature by providing new insights into the specific gendered challenges associated with regional resettlement [27]. This research identified that WoRB are particular vulnerable due to having limited English language proficiency, which makes accessing everyday basic needs and mainstream mental health services extremely difficult. The findings of the current study also challenge the current government’s plan to increase regional resettlement for refugee populations in Australia, and brings into question wither the needed preparatory work has occurred to ensure that regional resettlement locations can support the additional needs of this highly vulnerable population, with the findings of the current study suggesting that regional health services are currently under-resourced, under-funded and pushed to the extreme, leaving limited capacity for them to provide culturally sensitive support to the newest members of their community.

## Figures and Tables

**Table 1:** Initial Open-Ended Guiding Questions.

What are the key stressors for refugee women during resettlement?
What factors help WoRB cope during resettlement?
What are be biggest challenges for WoRB during resettlement?

## Data Availability

Due to ethical restrictions related to protecting the privacy imposed by the University of Tasmania and Charles Darwin University Research Ethic Boards, the full, qualitative dataset (transcripts and field notes) cannot be made public. Public availability would compromise participant confidentiality and privacy.
